# Label-Free Aptasensors for the Detection of Mycotoxins

**DOI:** 10.3390/s16122178

**Published:** 2016-12-18

**Authors:** Amina Rhouati, Gaelle Catanante, Gilvanda Nunes, Akhtar Hayat, Jean-Louis Marty

**Affiliations:** 1 BAE Laboratory, Université de Perpignan Via Domitia, 52 Avenue Paul Alduy, Perpignan 66860, France; a.rhouati@ensbiotech.edu.dz (A.R.); gaelle.catanante@univ-perp.fr (G.C.); 2 Ecole Nationale Supérieure de Biotechnologie, Constantine 25100, Algeria; 3 Technological Chemistry Department, Federal University of Maranhão, CCET/UFMA, Av. Portugueses, Cidade Universitária do Canga, 65080-040 São Luis, Brazil; gilvanda.nunes@hotmail.com; 4 Interdisciplinary Research Centre in Biomedical Materials (IRCBM) COMSATS Institute of Information Technology (CIIT), Lahore 54000, Pakistan

**Keywords:** aptamer, label free detection, mycotoxins, conformational changes, current trends

## Abstract

Various methodologies have been reported in the literature for the qualitative and quantitative monitoring of mycotoxins in food and feed samples. Based on their enhanced specificity, selectivity and versatility, bio-affinity assays have inspired many researchers to develop sensors by exploring bio-recognition phenomena. However, a significant problem in the fabrication of these devices is that most of the biomolecules do not generate an easily measurable signal upon binding to the target analytes, and signal-generating labels are required to perform the measurements. In this context, aptamers have been emerged as a potential and attractive bio-recognition element to design label-free aptasensors for various target analytes. Contrary to other bioreceptor-based approaches, the aptamer-based assays rely on antigen binding-induced conformational changes or oligomerization states rather than binding-assisted changes in adsorbed mass or charge. This review will focus on current designs in label-free conformational switchable design strategies, with a particular focus on applications in the detection of mycotoxins.

## 1. Introduction

Biosensors have emerged as a cheap and quick alternative to traditional chromatographic methods in the analytical assayfield. Biosensors are analytical tools which rely on the integration of bio-recognition molecules in the construction of sensor design. The most commonly employed bio-receptor elements in the biosensor application domain include enzymes, antibodies and aptamers [1,2]. However, the enzymatic biosensor field suffers from various drawbacks and their real time use is limited to certain specific applications. Several factors such as conformational change in amino acids at the active site of the enzyme can induce dramatic changes in enzymatic activity and substrate specificity, and subsequently influence the stability of the sensing enzymatic reaction. Enzyme denaturation is a property of vital significance in the fabrication of enzymatic biosensors. Enzyme denaturation can be induced by changes in pH, temperature, pressure, exposure to UV radiation, detergents, organic solvents or certain chemicals. The enzyme isolation process and subsequent incorporation into an in vitro operating environment can result in a loss of the enzymatic activity [3]. Though every enzyme has a set of specific optimum working conditions, but still a lot of research has been focused on how to improve the enzymatic stability over a long period of time [4]. As an alternative to enzymatic assays, one of the attractive candidates is immunoassays, likely due to the high affinity interactions between antigens and antibodies, often allowing higher sensitivity and lower limits of detection. However, antibodies are also proteinic in nature, and are prone to denaturation phenomena under varying experimental and physiological conditions. Moreover, they are mainly produced in living animals, and this results in relatively very high assay costs [5]. Phage display technology is proposed for in vitro selection of monoclonal antibodies characterized by high specificity and affinity. This technique is based on the genetic engineering of bacteriophages and repeated rounds of antigen-guided selection and phage propagation. However, heavy and light chain pairing may not reflect that of in vivo immunoglobulin [6]. In this context, a new class of molecules, named aptamers, has appeared as promising recognition tools for analytical applications. Aptamers are short single stranded oligonucleotides, either DNA or RNA, that fold into well-defined 3D structures and bind to their ligand by complementary shape interactions, with antibody-like binding ability. Aptamers present significant advantages over antibodies. As they are chemically synthesized, their production does not require the use of animals and is therefore less expensive and tedious. Aptamers can be also easily labeled with a wide range of reporter molecules such as fluorescent dyes, enzymes, biotin, or aminated compounds, enabling the design of a variety of detection methods [7]. Furthermore, the function of immobilized aptamers can be easily regenerated and aptamers can be reused. Due to these advantages, aptamers can thus be considered as a valid alternative to antibodies or other bio-receptors, in developing various analytical techniques. Various format assays using aptamers as bio-recognition elements have been reported in the literature. The conventional enzyme-linked aptamer assays, however, are time consuming, expensive and involve multistep processes. As aptamers and antigens are chemically inert, a label such as an enzyme is required to generate an electrochemical signal [6,7,8]. Although these developed methods are very sensitive, however, there are several challenges in the construction of labeled aptasensors. Labeling of either antigen or aptamer makes the assays more complex, time consuming and laborious. Moreover, the labeling process is costly and often results in the denaturation of the modified biomolecules [9]. In an effort to overcome these drawbacks, there is increasing interest in the development of label-free aptasensors.

On the other hand, since many small target analytes such as mycotoxins are present at low-levels, there are increasing demands for ultrasensitive detection methods for such molecules in the agro-food domain. High sensitivity is required because of the low maximum permissible levels of mycotoxins established by the European Commission. For example, ochratoxin A amount should not exceed 2 µg/Kg in wines and coffee and 5 µg/Kg in cereal products [8], while the limits for aflatoxin B1 in different foodstuffs have been set between 0.05 and 20 µg/Kg [9]. However, it is difficult to achive ultrasensitive detection of small molecules by a basic aptasensor design because of the very small size of the analytes. This problem has resulted in the exploration of novel aptasensor methods and designs to carry out the sensitive detection of mycotoxin analytes. Many methods for signal amplification in aptasensor design have been demonstrated, such as rolling circle amplification [10], strand displacement amplification [11] and enzyme label [12]. Although these methods offer advantages in terms of signal amplification, but are complicated, expensive and their operating conditions are rigorous [13]. Besides, aptamer-functionalized nanoparticles have been reported to amplify the signal in the development of aptasensors [14]. Using nanoparticles might overcome some of the problems, but still sensitive and in consistent methods are strongly desirable in the development of aptasensors. Based on the above observations, the objective of this review paper was to compare label and label-free methodologies, formats and detection techniques in the label-free aptasensor field, and finally mycotoxin detection as an example of small molecule analysis based on the label-free aptasensing methodologies is described in detail.

## 2. Why Label-Free Detection?

### 2.1. Labeled vs.Unlabeled Screening of Biomolecular Interactions

The use of labels to report bio-recognition events is very common in the development of detection strategies employed in different fields. The nature of the used molecule can vary widely; the label can be a radioactive or a fluorescent dye [15,16], metal complex or nanoparticles [17], enzyme with a detectable product … etc. [18]. This label is routinely attached to the target molecule or the bioreceptor (For example: aptamer or antibody). Analysis is then achieved by measuring the label activity or by the change in other chemical or physical properties on the transducer surface. The easy conjugation and convenient detection are the most important characteristics of a marker molecule. These features are required to ensure signal amplification for enhancing the sensitivity and selectivity of the method. Despite the advantages of label-dependant technology, the labeling and immobilization steps are time consuming and expensive. Indeed, non-specific adsorptions and luck of affinity between the labeled receptor and its target could be observed. This change in the binding properties can reduce the reproducibility, sensitivity or selectivity of the biosensor [19]. In addition to these drawbacks, online monitoring is not possible with labeled detection methods.

**Figure 1:**
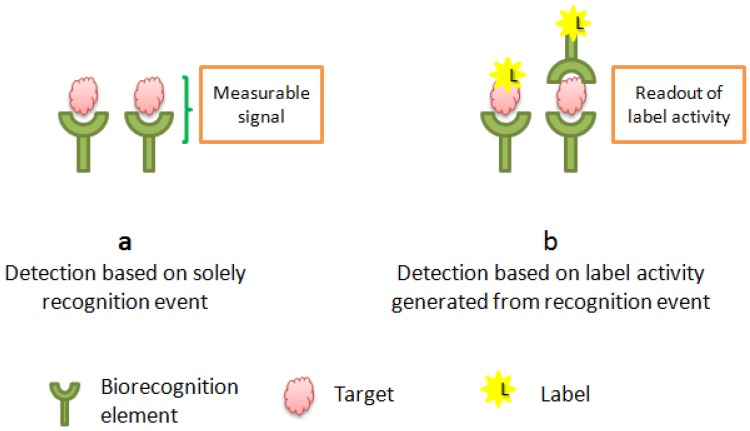
Label free (**a**) *vs*. label dependent biosensors (**b**).

Recently, label-free technology has emerged as an important strategy to allow the study of biomolecular interactions in real time. By avoiding the laborious labeling steps and the challenging label reaction/operations, the cost of the biosensor is reduced and the analysis can be performed within a shorter time. In this kind of operation, both partner molecules (target and bioreceptor) are not modified; they are used in their natural form. The bioreceptor is immobilized onto the transducer surface, and the sample containing the target is directly incubated with the functionalized surface. Analysis is then performed by studying the change in electrical or physical properties of the surface which depend solely on the affinity of interaction between the analyte and its receptor and thus the concentration of the analyte in the sample. The use of label-free monitoring increases the retention of the high affinity and decreases non-specific adsorptions [20]. Figure 1 shows the principal differences between labeled and non-labeled biosensors.

### 2.2. Label Free Detection Mechanism

In contrast to conventional biosensors, constituted of three essential parts—a bioreceptor, a signal reporter and a measurement instrument—label-free biosensors are only based on a bioreceptor and a physicochemical detector of the surface property changes. For that, the bioreceptor is first immobilized on a transducer surface, whose properties will be altered upon binding of the analyte. It is noteworthy that the affinity of the recognition element influences the biosensor’s specificity, while the immobilization strategy used has an important impact on the sensitivity [21]. The bioreceptor can be an enzyme, antibody, nucleic acid, microorganism, cell or a tissue.

Label-Free technology is based on the measurement of the generated signal obtained after the surface change induced by the analyte. It offers direct information about the interaction of the target with the sensing element by measuring changes on physical properties such as mass, refractive index, or electrical resistivity produced by this binding [22]. This change can be monitored by employing various detection mechanisms: electrical, optical or mechanical. In the first category, we study the conduction, capacitance or resistance, while in optical devices, changes in light (absorption and emission) are evaluated, and finally, mechanical sensors are mass and frequency-sensitive devices [23]. In addition to methods based on solid supports, label-free assays can also be performed directly in solution and are mostly coupled to optical detection.The best example of these assays are sensors based on the switchable structure of aptamers, aptamer DNAzymes and/or chemical of physical properties of nanoparticles [24]. Bulbul et al. reported an optical aptasensor based on the alteration of the catalytic activity of redox active nanoceria and the conformational change of OTA aptamer upon target binding [25]. The common point of these techniques is the relationship between the measured signal and the concentration of the bound target. The signal generated from the minimum detectable change corresponds to the limit of detection (LOD), while the dynamic range is related to the minimum and the maximum measured levels. Label-Free detection offer new opportunities to food safety field due to their numerous advantages such as: high sensitivity, simplicity, and possible miniaturization and portability, which are indispensable for point of care applications [22].

## 3. Aptamers in Label-Free Biosensing

Increased attention has been recently given to aptamer-based strategies and particularly, unlabelled assays. This returns to the numerous advantages of these promising recognition biomolecules over the traditionally used ones. Aptamers are synthetic, single or double stranded, oligonucleotides selected in vitro by Systematic Evolution of Ligands by EXponantial Enrichment (SELEX) technology for their ability to recognize a target [26]. In addition to their specificity and selectivity, the chemical nature and synthesis of aptamers make them cost effective and stable under extreme conditions. For that, aptamers have rivalled antibodies whose synthesis requires animal immunization which is expensive and time consuming. Indeed, because of their proteic nature, antibodies are not stable to certain pH and temperature variations [7,27].

Label-free aptasensing depends on smart interactions between an aptamer, its complementary strand, target or a signal probe. This unique property of nucleic acids allows the label-free sensing of a wide variety of targets, including small molecules, while the label-free immunosensing of such molecules requires more time and efforts [28].

### 3.1. Label-Free Aptasensing Formats

The formation of aptamer-target complex induces different types of changes where the aptamer can undorgo configurational or conformational modifications. Depending on this change label-free aptasensing strategies can be classified into: (1) structure switchable aptamer assays; (2) aptamer construct assembly/disassembly based assays and (3) target-induced variation in charge transfer transistance.

#### 3.1.1. Structure Switchable Aptamer Assays

This category of assays exploit the most important advantage of aptamers which consists in their switchable structure conformation upon the target binding. The ability of aptamers to form Watson-Crick base pairs, allows the replication of their primary structure onto secondary and tertiary structures. In contrast to the secondary structure, other base parings are implemented in the target induced-three dimensional folding of aptamers, where the paired and unpaired regions create appropriate structures with which the aptamer can form the ideal fit and bind tightly to its appropriate ligand [29]. Different three dimensional structures contributing in aptamer stabilization can be encountered, such as; G-quadruplex, hairpins and stem-loops [30,31].

**Figure 2:**
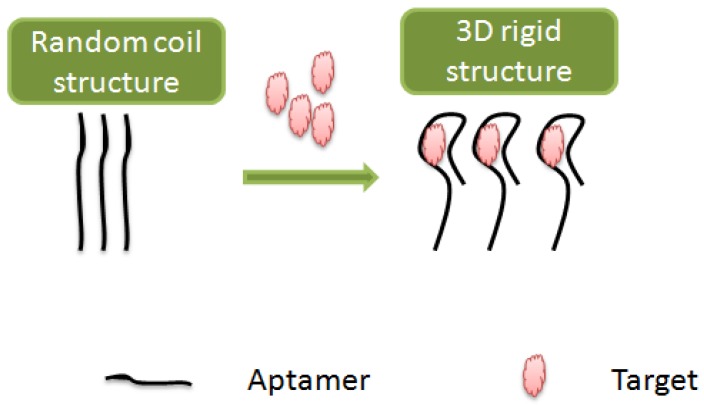
Principle of structure switchable aptamer-assays.

In aptaswitching detection strategies, aptamers are first folded into a stable three dimensional structure involving weak and non-covalent bonds; hydrogen bonding, van der Waals interactions and hydrophobic effects [32]. Then, the switchable event ocurrs by aptamer destabilization or attachment to a complementary strand. Finally, the conformational change is directly translated into a measurable signal (Figure 2) [33]. Based on this principle, many aptamer-based assays have been reported in the literature in labelled and unlabelled detection formats compatible with various types of transducers and target compounds [34,35,36,37]. This flexibility returns to the numerous advantages of this category of biosensors; the rapid response which can be obtained within few minutes, simple operation without the need of multiple transduction steps, reusability and reagentless structure switching [38,39].

#### 3.1.2. Aptamer Construct Assembly/Disassembly Based Assays

**Figure 3:**
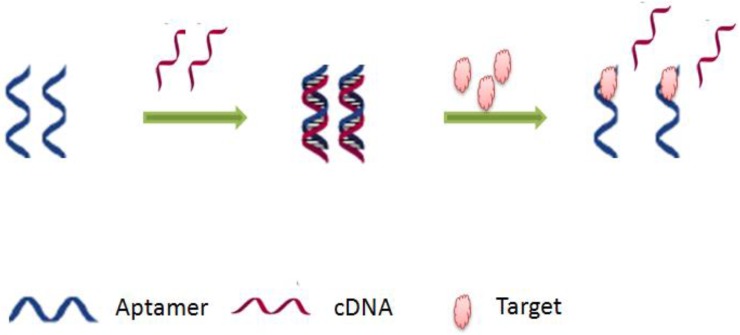
Principle of target-induced strand displacement aptasensing.

This category of label-free aptamer-assays is based on the modification of the sensor’s configuration. After formation of the aptamer-target complex, an association or a dissociation of the biosensing construct is induced. The association or assembly leads to the formation of a sandwich structure with a secondary aptamer. On the other hand, the dissociation or disassembly results in the release of a DNA strand, this strategy is also called target-induced strand displacement [40]. Both mechanisms cause changes on the surface electrode by generating a signal that can be translated by different detection methodologies (Figure 3) [38]. Several strategies have been developed by using this principle but mostly strand-displacement-based aptasensors [41,42,43]. In the usual design, aDNA double helix is immobilized on the electrode surface, in which one of the strands contained the aptamer sequence. Upon the target binding, the complementary strand is dissociated generating thus a detectable response. By comparison with structure switchable aptamer-assays, this strategy can be generalized, because it does not require a prior study of the secondary or tertiary structure of the aptamer [44].

#### 3.1.3. Target-Induced Variation in Charge Transfer Resistance

**Figure 4:**
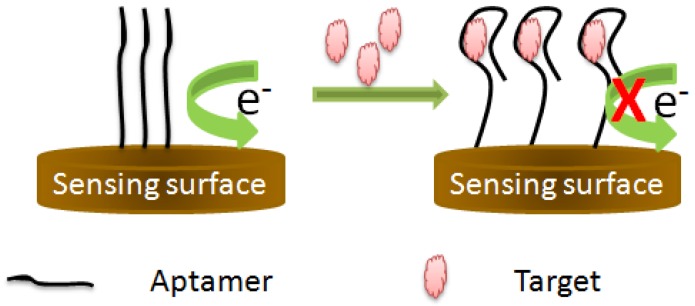
Principle of target-induced variation in charge transfer resistance.

This aptasensing format is the simplest one, because it is based on the direct monitoring of the interaction between the aptamer and its target. It does not depend on a conformational change of the aptamer structure or configurational change of the aptamer construct. In this kind of detection scheme, the binding of the target forms a resistant barrier on the sensing surface blocking thus the electron transfer to the electrode [45]. This change in charge resistance is proportional to the amount of the target in the sample. The charge transfer resistance (*R_ct_*) is a manifestation of the energy potential associated with the oxidation or reduction event on the surface, and the energy barrier of the redox species reaching the electrode due to electrostatic repulsionor steric hindrance [19] (Figure 4). Based on this principle, different aptasensing schemes have been reported for the monitoring of various targets [44,46].

### 3.2. Aptamer Immobilization Techniques

In an aptasensing scheme, the biological recognition can be analysed in solution or by immobilizing the aptamer onto a solid support. However, the immobilization technique used should maintain the affinity and specificity of the aptamer to its target. On the other hand, it should allow an easy recovery of the aptamer for multiple regenerations. For that, the aptamer can be physically adsorbed on the surface, covalently attached with functional groups or coupled to SAMS. Different reviews have discussed the surface immobilization methods of aptamers that we summarize in Table 1 [47,48,49].

### 3.3. Detection Methodologies in Label-Free Aptasensing

As mentioned above, label-free aptamer-based assays are adaptable with several transduction platforms which are sensitive to changes in interfacial properties, we will focus in this review on the most frequently used techniques; optical, electrochemical and mechanical detection methodologies.

#### 3.3.1. Optical Detection

In an optical biosensing scheme, the interaction of the biocomponent with the target molecule produces a change in the optical contents of the reflected light. This change is then detected by the transducer allowing the direct quantification of the target in a given sample [50]. Several types of optical methods can be used, among which colorimetric and fluorescence ones have gained more attention.

##### Colorimetric Assays

Colorimetric assays are based on a colour change which occurs in the presence of the target. The significant advantage of this kind of assay systems is the minimal need for special analytical instrumentation. In some cases, the results are visible by the naked eye and understandable without a priori knowledge. This unique property of colorimetry makes this technology a universal detection method for rapid, low cost and real-time monitoring of a wide variety of analytes [28]. These sensors often involve metal nanoparticles or DNAzymes (aptamers with catalytic activity) for signal amplification.

Gold nanoparticles (AuNPs) are excellent colorimetric indicators that have been widely associated to aptasensing schemes. The principle of these techniques is based on the aggregation/dispersion of AuNPs, determined by the presence or absence of salts, in addition to the key role of DNA in AuNps stabilization against salt-induced aggregation.The presence of the target in aptasensor system induces the nanoparticles re-aggregation. This property change from dispersion to aggregation leads to a colour change from red to blue which is proportional to the amount of analyte in the sample [17].

G-quadruplex-hemin DNAzymes have been also used for signal amplification in several label-free aptasensing stragies [51,52]. It is well established, that G-rich oligonucleotides can fold intro G-quadruplex structures stabilized by different metal ions. Certain of these structures are able to bind to hemin (a peroxidase cofactor) with high specificity and affinity. The formed complex G-quadruplex-hemin can function as a kind of peroxidase-mimicking DNAzyme. It has been demonstrated that the catalytic activity of this complex is much higher than that of hemin itself [53]. Based on these finding, an aptasening platform can be constructed by either releasing or blocking the G-rich DNAs that could form effective hemin-DNAzyme after targeting [28].

##### Fluorescent Assays

Since standard aptamers and most analytes do not have intrinsic fluorescent properties, fluorescent biosensors are based on the transduction of the biorecognition event into optical signal by employing small organic dyes as signal reporters [35]. Most of the label-free fluorescent aptasensors are based on molecular beacon strategy. A basic molecular beacon is a hairpin-shaped oligonucleotide with quenched fluorescent marker whose fluorescence is restored by the targeting [54]. In label-free assays, the principle of molecular beacon strategy is based on the enhancement of the dye’s fluorescence intensity by the aptamer, and its quenching upon the target binding. In aqueous solution, the signal reporter has a very low fluorescence emission, while the addition of the aptamer induces a remarkable change in the fluorescent properties. This phenomenon has been explained by Du et al., based on the fact the DNA can serve as a negative pocket to protect the fluorescent ligands from aggregation and the emission quenching in aqueous media [28]. Upon formation of the target–aptamer complex, the target changes the aptamer conformation and blocks the binding site for dyes. The dye is thus released with an obvious fluorescence decrease. Based on this fluorescence change, the concentration of the analyte in the sample can be measured. Although its sensitivity and specificity, this strategy is limited to aptamers exhibiting a target-induced conformational change. For that, a modified format has appeared as alternative; “duplex to complex” detection strategy can be performed, in certain cases, without aptamer conformational change. The aptasensing is based on the transition between aptamer duplex and ligand-aptamer complex [20].

#### 3.3.2. Electrochemical Detection

In a typical electrochemical aptasensor, the aptamer is immobilized on an electrode surface, while the recognition event is studied based on electrical current variations. As in label-free fluorescent assays, redox probes are bound to the aptamer through weak interactions such as electrostatic adsorption and hydrogen bonds. The target-induced change in the reporter’s electrochemical characteristics depends on the amount of the analyte in the sample. Based on the oxidation of aptamer bases, direct electrochemical detection is also possible. This can be accomplished by monitoring the oxidation of guanine and adenine which are the most redox-active bases [55]. Electrochemical Impedance Spectroscopy (EIS) and amperometric methods are widely used in label-free electrochemical aptasensing strategies.

In a label-free amperometric/voltammetric biosensor, the principle is based on the measurement of the current generated from the oxidation and reduction of an electroactive species at a fixed or variable potential. The change in the current corresponds to the concentration of the analyte in the sample. The amperometric detection can be direct if the target is potentially active, or indirect based on the redox reaction of an electroactive probe.

EIS allows the sensitive monitoring of changes in conductivity/resistance, or charging capacity of an electrochemical interface. The modification of a conductive surface, by a recognition element, induces the formation of an electrical double layer and a change in the resistance of the electrode-electrolyte interface. This unique property of impedance technology allows the effective and sensitive probing of affinity binding events [30]. Therefore, based solely on the resistance change, it is possible to monitor the formation of the complex aptamer-target directly independently to conformational change or strand displacement. Impedimetric biosensors can be Faradic or non-Faradic; depending on whether a redox probe is required or not. Non-Faradic impedance is experimentally simpler, because it does not involve redox processes, thus allowing practical real time monitoring [23]. In general, in both cases, Faradic and non-Faradic impedimetric biosensors, an increase in resistance is observed after the biorecongnition event. Taking as example, a Faradic strategy where the anionic [Fe(CN)_6_]^3−/4−^ couple is used as redox probe, in this case, the formation of the complex aptamer-target blocks the redox probe from approaching the electrode surface thus increasing the resistance. In addition to standard methods, impedimetric label-free aptasensing, based on strand displacement can be also performed. For that, the aptamer is bound to a complementary strand to form a duplex. After the target-binding, the aptamer is displaced from the duplex into solution, with a corresponding decrease in electron transfer resistance [44]. Despite their sensitivity, selectivity and low cost, label-free impedimetric biosensors still suffer from some limitations such as luck of reproducibility. Theorically, the electron-transfer rate is selectively modulated by the analyte [56], however, impedance can be affected by the immobilization technique adopted for the surface functionalization. For example, the ability of the functional groups to be ionized may have an effect on the charge transfer, influencing thus the assay reproducibility [19].

#### 3.3.3. Mechanical Detection

Despite the predominance of optical and electrical detection methods in the realm of biosensors, the increasing knowledge about the mechanical nature of biological mechanisms has led to the development of promising mechanotransuction processes to detect biological interactions [57]. Piezoelectric materials (ex; quartz) and cantilevers are the most commonly used transducers to monitor the mass change resulting from the recognition events.In a cantilever-based biosensor, the target binding is translated into defelection or changes in the resonance frequency of a cantilver which reflects on the analyte amount in a sample [58], whereas, in quartz-cristal microbalance (QCM) biosensors, the biocomponent is immobilized on a quartz material which is a stable and sensitive oscillator. The biochemical reaction decreases the oscillation frequency of the quartz cristal of the microbalance allowing thus the quantitative detection of the mass deposited on the electrode surface [45,59].

## 4. Label-Free Aptasensors for Myctotoxin Determination

Mycotoxins are secondary fungal metabolites of low molecular weight that contaminate food and feedstuffs. They occur more frequently in areas with a hot and humid climate, favorable for the growth of molds, but they can also be found in temperate zones [60]. Mycotoxins pose serious health and economic problems, being responsible for mycotoxicosis, at low levels, with symptoms of intoxication causing substantial effects on animal and human health [5]. Among about 200 known mycotoxins, ochratoxins, aflatoxins and fumonisins are the most toxic [61]. Aptamers and aptasensors reported in the literature have been mostly designed for ochratoxin A (OTA), aflatoxins B1, B2 and M1 (AFB1, AFB2 and AFM1), fumonisin B1 (FB1) and zearalenone (ZEA). Table 2 summarizes the different label-free aptasensors reported in the literature for mycotoxins determination.

### 4.1. Label-Free Aptasensors for OTA Detection

**Figure 5:**
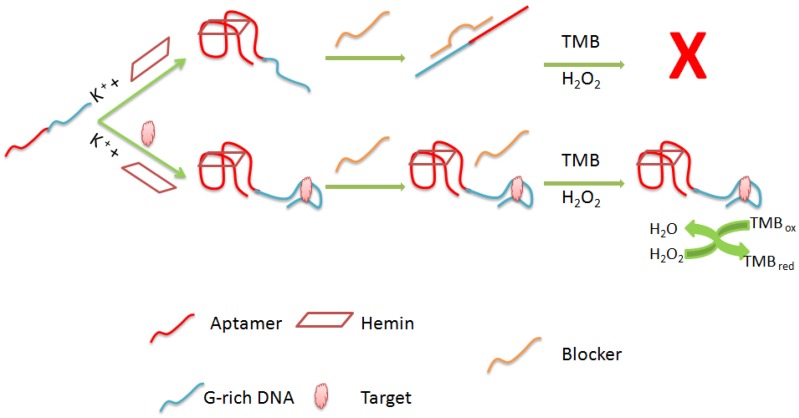
Principle of a colorimetric aptasensor based on HRP-mimicking DNAzyme. Incubation of the aptasensor with OTA reduces the affinity between the first oligonucleotide (aptamer+DNAzyme) and the second one which is the blocker increasing thus the HRP activity [53].

Because of its widespread occurrence and hazardous effects on animal and human health, OTA is the most studied mycotoxin in food analysis. Produced by several fungalspecies of the genera *Penicillium* and *Aspergillus*, OTA contaminates a wide variety of food matrices, particularly cereals, grape products and roasted coffee [88]. Since the selection of OTA’s aptamer by a Canadian group in 2008, various labeled and unlabeled aptasensing strategies have been reported [89]. Despite the recent advances in label-free biosensing, OTA determination using unlabeled aptasensors is still a challenge. Our lab has reported a colorimetric assay based on a horseradish peroxidase (HRP)-mimicking DNAzyme whose advantages have been discussed above. DNAzymes present a promising alternative to the use of enzymes in aptamer-based biosensors. The label-free aptasensor was designed using two oligonucleotides. The first comprises OTA-aptamer and DNAzyme sequences (the principle is shown in Figure 5). The second one consists in a blocking DNA which includes a partially complementary sequence to a part ofthe aptamer and partially complementary one to a part of the DNAzyme. The principle of the bioassay is based on the target-induced DNA folding to form a rigid antiparallel G-quadruplex structure. This conformational change inhibits the hybridization of the two oligonulceotides thus increasing the catalytic activity of the HRP-mimicking DNAzyme. The aptasensor was applied on wine samples and the spectrophotometric measurements showed a linear correlation between DNAzyme activity and OTA concentration in the range of 3.6–120 µg/L. However, the LOD of this assay was higher than the maximum tolerated level of OTA in wines established by the European Commission (2 µg/L) [62]. Wang et al. combined the advantages of G-quadruplex DNAzymes and hybridization chain reaction technology (HCR). HCR is a signal amplification technique, based on the chain reaction of recognition and hybridization events between two DNA hairpin molecules. The aptasensor was designed by using two hairpin oligonucleotides, the aptamer is localized in the 3’-end of the first molecule while the DNAzyme is in the central part of the second hairpin. The target binding induces the opening of the hairpin structure and the hybridization chain reaction releasing thus many DNAzymes. This HCR generates enhanced colorimetric signals, which is correlated to the OTA concentration in the sample. The reported aptasensor showed a high sensitivity (LOD of 0.004 µg/L) [63].

Recently, Bulbul et al. reported a colorimetric method based on the conformational transition state of the aptamer on nanoceria, combined with the redox properties of these particles. Changes in the redox properties at the nanoceria surface upon binding of the ssDNA and its target analyte enables rapid and highly sensitive measurement of OTA. By binding different aptamer concentrations with a fixed OTA concentration, the association constant of aptamer-OTA conjugate on nanoceria was calclulated as 0.120 nmol^−1^. In addition, the authors have shown that the optical properties of nanoceria are conserved and enhanced after 2 days [25].

Simple label-free fluorescent aptasensors have been also reported for OTA analysis to overcome fluorphore-labeled aptamers limitations. These strategies are mostly based on an enhancement in the fluorescence intensity of a dye by intercalation with an aptamer. First, Zhang et al. reported a fluorescent aptasensor based on the ability of ssDNA to enhance the emission of terbium ion(Tb^3+^) in solution. The aptasensor was constructed by immobilizing OTA aptamer on magnetic beads (MBs). In absence of OTA, the aptamer hybridized with two ssDNA probes present in the solution, blocking thus the Tb^3+^ emission. Upon addition of OTA, the aptamer structure switched to G-quadruplex and released the ssDNA probes resulting in an enhancement in Tb^3+^ fluorescence in solution proportional to the amount of OTAwith a limit of detection as low as 0.02 µg/L [64]. In contrast to Tb^3+^, Pico Green reagent (an asymmetric cyanine dye) fluoresces upon binding to dsDNA, while ssDNA does not change its fluorescence intensity. Based on this principle, Lv et al. developed a fluorescent label-free aptasensor for OTA determination in beer samples. The detection was based on a competition for aptamer between aptamer/OTA complex and aptamer/cDNA duplex, where, the quantity of OTA was inversely proportional to the fluorescenceintensity in the wide linear range of ( 1 to 100,000 µg/L) [65]. Furthermore, McKeague et al. used another fluorescent dye (SYBR green) whose fluorescence is enhanced upon binding to dsDNA. Increasing concentrations of the OTA caused a displacement of SYBR green from the aptamer, and a concentration-dependent loss of emitted fluorescence. In this work the authors selected a new specific aptamer for OTA that showed similar characteristics to the original one. However, the aptamer shows modest affinity to OTB and negligible response to wafarin [66]. In addition to fluorescent probes, luminescent probes were also investigated in label-free aptasensing of OTA. Lu et al. used luminescent metal complexes (octahedral Iridium(III)) selective for G-quadruplex structures to construct a switch-on aptasensor. Two oligonucleotides have been used; the first comprising OTA aptamer, hybridized with a partially cDNA strand. OTA binding induced the disassembly of the duplex, allowing the aptamer to fold into a quadruplex structure and adecrease of luminescence. The authors suggested that this duplex to complex approach is advantageous compared to a random coil-to-quadruplex strategies, because of the resistance of initial duplex substrate against the presence of interfering ionswhich may induce aptamer folding even without the addition of OTA [67].

Despite the sensitivity of the mentioned optical aptasensors, their reusability was not demonstrated. Park et al. developed a regenerable label-free localized surface plasmon resonance (LSPR) aptasensor based on a structure switching aptamer and gold nanorods. The detection of OTA was achieved by monitoring the change in the magnitude of the LSPR wavelength which depended on the location of the analyte relative to the surface of the nanoparticle and the degree of alteration of the refractive index. Importantly, the biosensor was regenerated by heating in methanol at 70 °C [68].

EIS is the most employed method for label-free aptasensing of OTA in food samples. This is due to the high sensitivity, low cost, fast response time and simple equipment. Aptamer immobilization and the oriented organization of biomolecules on the sensor play a key role in the performance of a biosensor. For that, several immobilization strategies have been reported in the literature for impedimetric label-free aptasensing. In 2011, Prabhakar et al. described the first impedimetric label-free aptasensor for OTA detection. In this report, OTA aptamer was covalently immobilized onto mixed Langmuir–Blodgett (polyaniline–stearic acid) film deposited onto indium tin-oxide coated glass plates. Then, the change in the magnitude of transfer resistancedue to OTA binding observed at the sensor surface is utilized for sensitive detection of OTA. The aptasensor is regenerable by disrupting aptamer-OTA complex in 50mMNaOH for 2min [69]. Despite its sensitivity (LOD of 0.1 µg/L), the fabrication procedure of this sensor required complicated immobilization steps as compared to the simple chemisorption reported by Castillo et al. In this work, the thiolated aptamer with different configurations was chemisorbed on the surface of a gold electrode. OTA determination was based on the monitoring of charge transfer resistance which increased with increasing OTA concentration in the sample. A redox probe [Fe(CN)_6_]^3−/4−^, was used to amplify the detection of the interaction aptamer-OTA. The sensor was validated with coffee, floor and wine samples with high sensitivity (LOD of 0.048 µg/L) [70]. Furthermore, we designed in our lab different label-free aptasensors for OTA determination by using innovative immobilization chemistries. In the first one, azido-aptamer was immobilized onto an electrografted binary film by click chemistry. After modification of ascreen printed carbon electrode (SPCE) surface with a layer of active ethynyl groups, the latter reacted efficiently with aptamer bearing an azide function in the presence of copper (I) catalyst. The increase in electron-transfer resistance values due to the specific aptamer–OTA interaction was proportional to the concentration of OTA where the LOD achived 0.00025 µg/L [71]. We have demonstrated that the immobilization *via* click chemistry improved the aptamer binding capacity and magnified the response signal of the aptasensor. For that, the same strategy was exploited in another report for on-site monitoring of OTA in cocoa beans and cocoa powder [72]. Later on, we proposed another immobilization strategy for label-free electrochemical OTA aptasensing. The sensor was based on two piece macromolecules; an aminomodified aptamer covalently attached to the carboxy end of a PEG (polyethylene glycol) spacer immobilized on a SPCE surface. In this work, the LOD was two fold lower than that obtained by using click chemistry (LOD= 0.00012). These macromolecules formed long tunnels on SPCE surface, while aptamer acted as gate of the tunnels. The principle of this aptasensor is based on targeted induced conformational changes, OTA binding closed aptamer gates, decreasing the electrochemical signal [73]. In addition to its high sensitivity, the reported aptasensor required less time and simpler operation as compared to the previously described aptasensors. The same principle was used by employing hexamethyldiamine instead of PEG spacer, while electrochemical detection of OTA was performed by cyclic voltammetry (CV) [22].

Nanomaterials have been also exploited in the development of impedimetric aptasensors because they present a promising tool for electrode surface modification. This is due to their numerous advantages such as good conductivity and large surface area. Evtugyn et al. attached a thiolated aptamer to AuNPs stabilized by a hyperbranched polymer; dendrimeric hydrophilic Boltorn H30^®^. The formation of OTA-aptamer complex induced the conformational switch of the aptamer from linear to guanine quadruplex leading to the consolidation of the surface layer and an increase of the charge transfer resistance [75]. Recently, iridium oxide (IrO_2_) NPs have been also used for aptamer immobilization; SPCE surface was modified with an electropolymerized film of polythionine followed by the assembly of IrO_2_ NPs. The amino-modified aptamer was subsequently exchanged with the citrate ionssurrounding IrO_2_ NPs via electrostatic interactions with the same surface. It is well established that electropolymerization improves the conductivity and provides a stable redox-active coatings on the electrode surface. The reported method exhibited the low LOD of 0.0056 µg/L and a linear range of (0.004–40 µg/L) [76]. The use of silver nanoparticles has been also reported for quantitative investigation of OTA using nano-impact electrochemistry. The principle of this method is based on the target-induced changes in collision frequency. These changes are assigned to the surface coverage of nanoparticles by the aptamer and the conformational change of aptamer which affected the electron transfer between the electrode and silver nanoparticles [76].

### 4.2. Label-Free Aptasensors for Aflatoxins Detection

Aflatoxins are toxic metabolites produced by filamentous fungi like *Aspergillus flavus* and *Aspergillus parasiticus*. They are present in agricultural products and animal feeds, including tree nuts, peanuts, peanut butter, figs and corn. They are responsible forserious human health disorders: hepatocellular carcinoma, aflatoxicosis, Reye’s syndromeand chronic hepatitis. Several types of aflatoxins are known and classified into six subtypes: aflatoxin B1, B2, G1, G2, M1 and M2, aflatoxin B1 is the most predominant and toxic class [78]. AFB1 aptamer was first selected and patented by Neoventures Biotechnology Inc. (London, ON, Canada) [90]. Then, Wang’s group identified specific aptamers for AFB1 and B2 [91], while AFM1 aptamer has been selected by Malhotra et al. [92]. After selection, these aptamers have been used, in many reports, as biorecognition elements in optical and electrochemical label-free aptasensing strategies.

Based on salt-induced AuNPs aggregation phenomenon, Luan et al. described two colorimetric label-free aptasensors for AFB1 and AFB2 detection. In the absence of the target, the nanoparticles were stabilized and dispersed by the aptamer leaving the solution red under high NaCl conditions. After that, the target-induced conformational change exposes the AuNPs to NaCl-induced aggregation leading to a colour change. The linear dynamic range and detection sensitivity were found to be 0.025–100 µg/L and 0.025 µg/L of AFB1, respectively [77,78]. Seok et al. reported another colorimetric assay based on AFB1-induced DNA structural changes and peroxidase mimicking DNAzyme. For that, the aptamer was combined with split halves of hemin-binding DNAzymes. AFB1 binding induced a structural deformation of the aptamer-DNAzyme complex, which caused splitting of the DNAzyme halves thus decreasing peroxidase mimicking activity and the colour signal in the wide linear range of 0.1–1.0 × 10^4^ µg/L [79]. Based on the same principle of HRP mimicking DNAzymes, a competitive chemiluminescent aptasensor has been developed for AFB1 detection in corn samples. In this work, the AFB1 aptamer linked with a dual HRP-DNAzyme produced sufficient chemiluminescence(CL) values when binding to AFB1-ovalbumin (OVA) used as a coating antigen. The assay was based on the monitoring of the CL produced from the interaction between the aptamer/HRP-DNAzymes and luminal. Analytical performances of the sensor have been validated on corn samples where the LOD attained 0.11 µg/L and the extraction recoveries averaged from 60.4% to 105.5% [80]. Recently, a label-free fluorescent aptasensor was developed for the simultaneous detection of OTA and AFB1 in cereals. The corresponding aptamers, hybridized with ssDNA signal probes, have been immobilized on MBs. After the target-induced strand displacement followed by magnetic separation, the released probes present in the supernatant acted as the corresponding scaffolds to synthesize silver nanoclusters with different photoluminescence emission bands. The authors have noted an increase in fluorescence intensity after adding Zn(II)-ion into the system. In addition to its sensitivity (LOD = 0.0002 µg/L), the reported method allowed the discrimination between the two mycotoxins in food samples [81].

**Figure 6:**
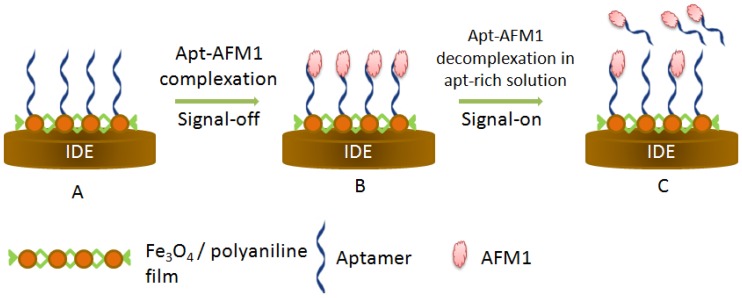
Principle of label-free aptasensor of AFM1 based on Fe_3_O_4_ /polyaniline film polymerization on interdigitated electrode. (**A**) Functionalization of the surface with AFM1 aptamer; (**B**) Signal-off experiment performed after target binding; (**C**) Signal-on detection achieved by treating the biosensor with aptamer rich solution which results in the displacement of some AFM1 that leave the electrode to go into the solution where aptamer concentration is higher [82].

Owing to their low cost, simplicity and high sensitivity, electrochemical aptasensors have been also applied successfully to aflatoxins. Nguyen et al.employed CV and square wave voltammetry to monitor the biomolecular interaction aptamer-AFM1. For that, the aptamer has been immobilized on Fe_3_O_4_ incorporated polyaniline film polymerized on interdigitated electrode (IDE). AFM1 binding strongly influenced the switching rate of polyaniline film and decreased the current, after blocking the charge transfer to the electrode surface, which is inversely proportional to the analyte concentration. The principle of the signal-on detection, shownin Figure 6, is based on the competitive reactions between AFM1-aptamer complex and free aptamer present in solution occurring by virtue of equilibrium displacement. The treatment of the electrode with an aptamer-rich solution induced the dissociation of aptamer-AFM1 complexes, where the released AFM1 left the electrode surface to the aptamer-rich solution. The described biosensor has shown high sensitivity (0.00198 µg/L), excellent stability and reproducibility. However, the applicability of the method for AFM1 determination in real samples was not demonstrated [82]. Recently, our research group reported an impedimetric aptasensor for AFM1 detection in milk samples. In this work, the aptamer, modified with hexaethyleneglycol, was covalently immobilized on a SPCE surface activated with diazonium salts. The method was successfully applied toreal milk samples with a good correlation with a conventional immunoassay [83]. Using the same immobilization strategy, we described an impedimetric aptasensor for AFB1 by comparing two different aptamers. Both aptasensors showed high sensitivity, reproducibility, fast response and good selectivity. The biosensors have been tested on alcoholic beverages with promising recovery percentages (92% to 102%) [84]. Another original immobilization platform was adopted by Castillo et al. to attach AFB1 aptamer for the development of an electrochemical aptasensor. The amino-modified aptamer was immobilized on gold electrode covered with cystamine-poly(amidoamine) dendrimers layer. The thickness of this layer decreased after AFB1 binding, this was explained by target-induced conformational change of aptamer. The electrochemical detection revealed that the current peaks decreased by increasing aflatoxin concentration in the dynamic linear range (0.03–3.125 µg/L). The reusability of the sensor was demonstrated by using 0.2 M glycine-HCl [85].

Finally, real-time quantitative polymerase chain reaction (RT-qPCR) can be also used as a detection method of biomolecular interactions. Guo et al. reported a simple and low cost aptasensor based on target-induced strand displacement. In this detection strategy, AFB1 aptamer was hybridized with its complementary strand which acted as a signal generator for PCR amplification. The aptasensing was achieved by monitoring the amplification signal which was related to AFB1 concentration. In addition to its simplicity (the whole sensing procedure was accomplished in a single PCR tube), the reported method israpid, low cost and highly sensitive (LOD = 0.000025 µg/L) [9].

### 4.3. Label-Free Aptasensors for Other Mycotoxins

**Figure 7:**
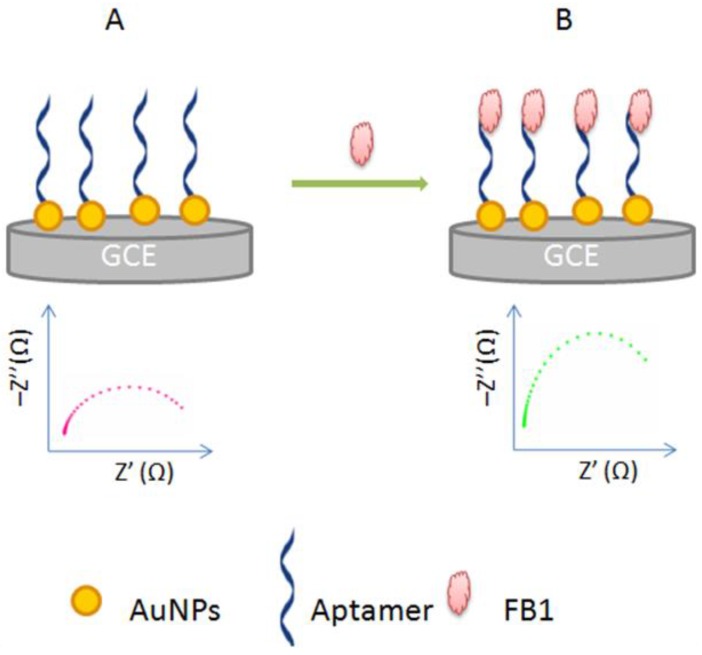
Principle of impedimetric aptasensor for FB1 detection. Niquist plots of the impedimetric aptasensor before (**A**) and after incubation with FB1 (**B**) [86].

Two aptamers have been selected by different research groups for the carcinogenic mycotoxin fumonisin B1 [93,94]. This mycotoxin, produced by *Fusarium moniliforme*, occurs mainly in maize and in processed maize products and animal feeds. After selection and characterization of FB1 aptamer, Wang’s group reported an impedimetric aptasensor for FB1 detection. The thiolated aptamer was anchored on AuNPs directly electrodeposited on a glassy carbon electrode (GCE). AuNPs have been used for their electrical conductivity and ease of self-assembly through athiol group. After the incubation of the aptasensor with FB1, the electron transfer between the [Fe(CN)_6_]^3−/4−^ electrolyte solution and the electrodewas significantly inhibited, resulting in a corresponding increase in the resistance monitored by EIS (Figure 7). Finally, the applicability of the method was proved on maize samples with satisfactory results: the extraction recoveries ranged from 91% to 105% [86].

Label-free mass sensitive aptasensors have been also investigated in myctotoxin determination. Chen et al.functionalized the sensing cantilevers in the array with self assembled monolayers (SAMs) of thiolated FB1 aptamer. Aiming to avoid interferences in the environment, reference cantilevers were modified with 6-mercapto-1-hexanol SAMs. Then , the analyte concentration was proportional to the diffential deflection amplitude between sensing and reference cantilevers with a low LOD (33 µg/L) [87].

The same research group selected a specific aptamer for the mycotoxinzearalenone, anon-steroidal estrogenicmycotoxin produced by *Fusarium graminearum* on maizeand barley [95]. However, to our knowledge there are no reports on label-free aptamer-based determinationfor zearalenone.

## 5. Limitations and Challenges of Aptasensors 

Despite the numerous advantages of aptamers as bioreceptors and aptasensors as promising analytical devices, they still suffer from some limitations and more efforts are needed to allow their successful applicability in complex samples. As they are nucleic acid biopolymers, thein situ application of aptamers is crucially limited by their inherent physicochemical characteristics. For overcoming that, various analytical parameters should be considered and predetermined during the selection procedure of aptamers. The target should be bound to the random oligonucleotide library in a selection environment with certain experimental conditions such as:temperature, pH, ionic strength and buffer components. These conditions are studied according to the sensing environment in which the target will be assessed. They contribute in the specific selection stringency improving thus the aptamer’s affinity and function [96,97].Besides these characteristics, selectivity plays a key role in the performance of an analytical device. Selectivity of aptamers can be enhanced by introducing a negative SELEX step. In this counter selection, the random library is incubated with analogue targets, excluding thus, the candidates exhibiting an affinity to these targets [98].

The possibility to realize aptamer selection under in vitro conditions opens the door for a wide range of post-SELEX modifications. It has been shown that post-SELEX modification can result in further enhancement of binding affinity and specificity, as well as other desired properties [99]. Indeed, post-SELEX modifications allow the bioconjugation of aptamers to transducers, labeling biomolecules and signal amplificators in order to enhance the aptasensor’s sensitivity.

Finally, although the promising advantages of aptamers over antibodies, immunoassays still substantially dominate the market. This is due to the limited number of aptamers available, whether for biomedical or food safety applications.

## 6. Conclusions and Future Prospects

Observing molecular binding events, especially for small size molecules, requires the design of methods that are sensitive to a very small amount of change in the interface of the sensor, to generate a detectable signal. To date, there are no general labelled sensing platforms that can be applied for very sensitive and selective detection of small size molecules for real time applications. Major limitations include, but are not limited to time consuming labelling steps, non-native signal interference, non-suitability for decentralized analysis, need of highly sophisticated and costly equipment, and the requirement forskilled persons to operate these equipment.

The development of label-free methodologies, however, has overcome these limitations and a success level is achieved in this direction to certain extent. Label-free methodologies output a signal that is directly related to the interaction of target analyte with the biomolecule, contrary to label methods where the signal is based on the label-attached molecules, and subsequently issues of false positive and labelling process can be avoided. Moreover, it is expected that label-free sensing methodologies are more reproducible as compared to those based on the labelling of molecules. The integration of aptamers as bioreceptor elements in the design of label-free biosensors has further improved the analytical performance of the assays. The long term stability and in vitro production of the aptamer molecule along with label-free detection methodologies makes these assays very cost-effective and highly suitable for field analysis. The phenomenon of aptamer conformational changes upon target analyte binding offers numerous advantages in the fabrication of label-free methods, as antibody-based label-free methods are based on the mass changes and label-free immunosensors are limited to a few assay formats. While aptamers offer additional formats such as those based on the signal on and signal off detection approaches. In this context, nanomaterials have been successfully integrated with aptamer to design label-free assays in solution. The electrostatic attachment or the simple adsoption of aptamer alters the optical properties of the nanomaterials, which are restored in the presence of target analyte. Similary alteration in the redox properties of nanoparticles upon aptamer conguation, and subsequent incubation of analyte have been used for the designed optical aptasensors. These assays permits very fast analysis of target analytes and have comparable analytical performance with those based on the label methodologies.

In this review paper, we have further shown that how these label-free aptasensor methods are successfully employed for the detection of the small size molecules of mycotoxins. Although, a great level of success has been achieved in the domain ofaptamer-based label-free detection of mycotoxins, however, there are still some gaps to be filled by researchers. For example, all types of mycotoxins are toxic to a certain level and their monitoring is equally important. However, most of the label-free aptasensors reported in the literature are employed for the detection of ochratoxin A and aflatoxin detection. Aptamers are not selected against all type of mycotoxins, future research may focus in this direction to develop new aptamer sequences for these mycotoxins. Similarly, most of these aptamer label-free assays are based on the electrochemical transducer platform or performed in solution. Filter paper has emerged as a very cheap and attractive optical transducer platform foron-site analysis of various contaminants which can be exploited in the design of label-free aptasensors for the detection of mycotoxins.

## Figures and Tables

**Table 1:** Different types of aptamer immobilization techniques for aptasensor applications.

Technique	Principle	Advantages	Limitations	Example of Bioconjugation
Physical adsorption	Electrostatic forces Van der Waals interactions	Simple and rapid	Weak attachment Random orientation of aptamers	Direct attachment on metals surfaces and surfaces coated with hydrophobic polymers
Covalent attachment	Interactions between the surface functional groups and aptamer’s chemical groups	Wide range of functional groups Flexibility	Multiple conjugation steps Non specific binding	NHS ester chemistry Click chemistry
SAMs	Amphiphilic molecules: hydrophilic and hydrophobic groups with respective affinity to the transducer and the aptamer	Stability Oriented recognition	More suitable with silicon and gold surfaces	Thiols and alkyne disulfides on gold Alcohols on glass

**Table 2:** Label-Free aptasensors for mycotoxin determination in food reported in the literature.

Mycotoxin	Detection	Assay Principle	Linear Range (µg/L)	LOD (µg/L)	Sample	Ref
OTA	Colorimetric	HRP mimicking DNAzyme	3.6–120	12	Wine	[62]
	Colorimetric	HRP mimicking DNAzyme, Hybridization chain reaction	0.004–0.96	0.004	Yellow rice, wine, wheat flour	[63]
	Colorimetric	Structure switching aptamer Nanoceria	0.08–12	0.06	Milk	[25]
	Fluorescence	Structure switching aptamer Tb^3+^, magnetic sepatation	0.1–1	0.02	Wheat	[64]
	fluorescence	Structure switching aptamer Pico green dye	1–100,000	1	Beer	[65]
	Fluorescence	SYBR green dye	3.6–40	3.6	-------	[66]
	Luminescence	Structure switching aptamer Iridium(III)	2–60	2	-------	[67]
	LSPR	Structure switching aptamer Red shift of LSPR band	0.4–400	0.4	Ground corn	[68]
	EIS	OTA-induced change in *R_ct_*	0.1–10	0.1	--------	[69]
	EIS	OTA-induced change in *R_ct_* [Fe(CN)_6_]^−3/−4^	0.04–40	0.048	Coffee, flour, wine	[70]
	EIS	OTA-induced change in *R_ct_* [Fe(CN)_6_]^−3/−4^	1.25 ×10^−3^–0.5	0.25 × 10^−3^	Beer	[71]
	EIS	OTA-induced change in *R_ct_* [Fe(CN)_6_]^−3/−4^	0.15–2.5	0.15	Cocoa	[72]
	EIS	Structure switching aptamer OTA-induced change in *R_ct_*	0.12 ×10^−3^–5.5 ×10^−3^	0.12 × 10^−3^	Beer	[73]
	CV	Structure switching aptamer OTA-induced change in *R_ct_*	0.12–8.5	0.1	Beer	[74]
	EIS	Structure switching aptamer OTA-induced change in *R_ct_*	0.04–40	0.008	Beer	[75]
	EIS	Structure switching aptamer OTA-induced change in *R_ct_*	0.004–40	0.0056	Wine	[22]
	Nano-impact electrochemistry	Structure switching aptamer OTA-induced collision frequency changes	0.028–4	0.02	------	[76]
AFB2	Colorimetric	Structure switching aptamer AuNPs aggregation	0.025–10	0.025	Beer	[77]
AFB1	Colorimetric	Structure switching aptamer AuNPs aggregation	0.025–100	0.025	-----	[78]
AFB1	Colorimetric	HRP mimicking DNAzyme	0.1–1.0 × 10^4^	0.054	Ground corn	[79]
AFB1	Chemiluminescence	HRP mimicking DNAzyme	0.1–10	0.11	Corn	[80]
OTA and AFB1	Fluorescence	Target-induced strand displacement DNA-scaffolded silver nanoculsters	0.001–0.05	0.0002 and 0.0003	Rice, corn, wheat	[81]
AFM1	CV, SWV	Target-induced blocking of chargetransfer to the electrode surface	0.006–0.06	0.00198	--------	[82]
AFM1	EIS	Target-induced change in *R_ct_*	0.002–0.15	0.00115	Milk	[83]
AFB1	EIS	Target-induced change in *R_ct_*	0.125–16	0.12	Beer and wine	[84]
AFB1	CV, EIS	Target-induced blocking of chargetransfer to the electrode surface	0.03–3.125	0.125	Peanuts	[85]
AFB1	RT-qPCR	Target-induced strand displacement	5 × 10^−5^–5	0.000025	Chinese wildrye hay and infant rice cereal samples	[9]
FB1	EIS	target-induced change in *R_ct_*	72–720 × 10^3^	1.44	Maize samples	[86]
	Microcantilever	Target-induced change in surface stress	33	100–40,000	----------------	[87]
